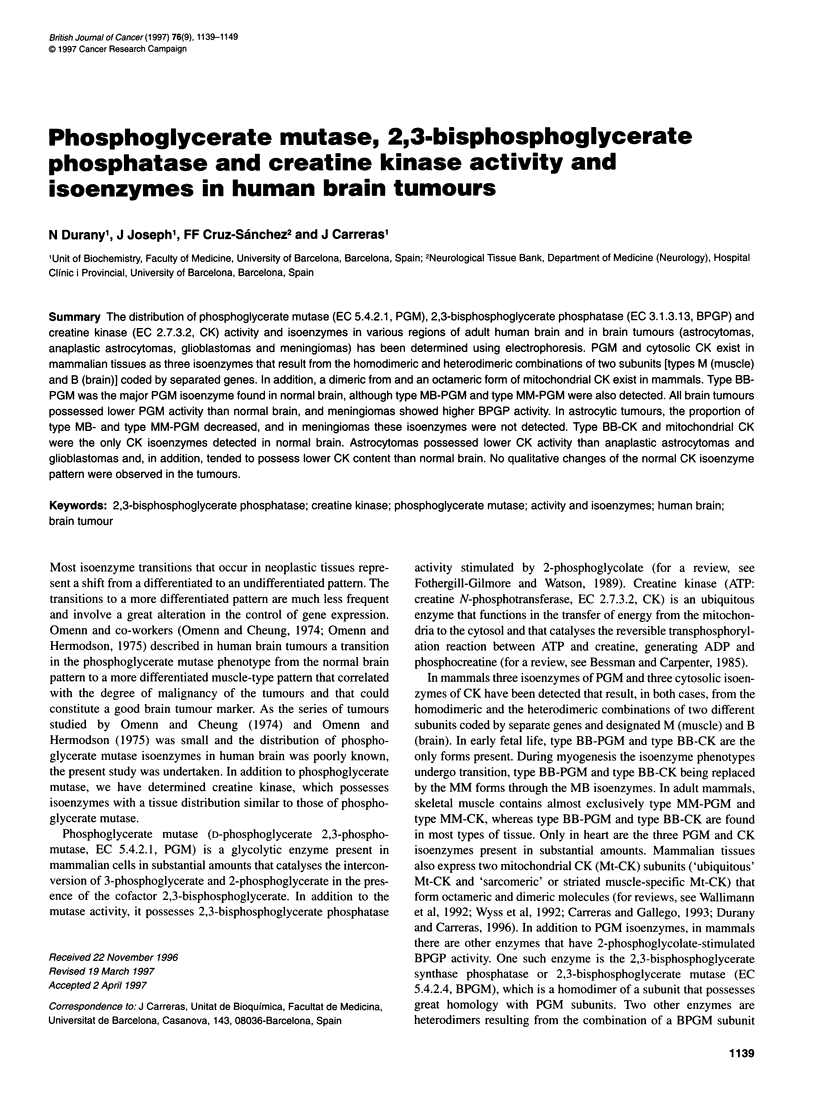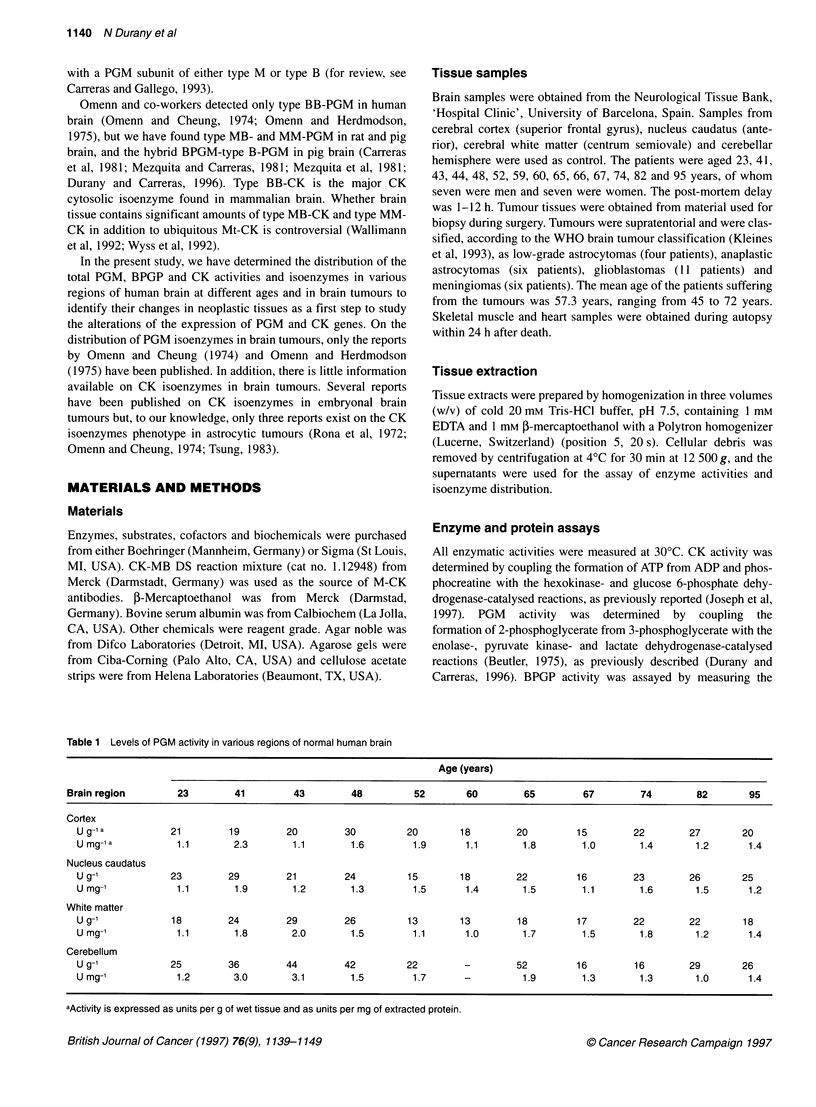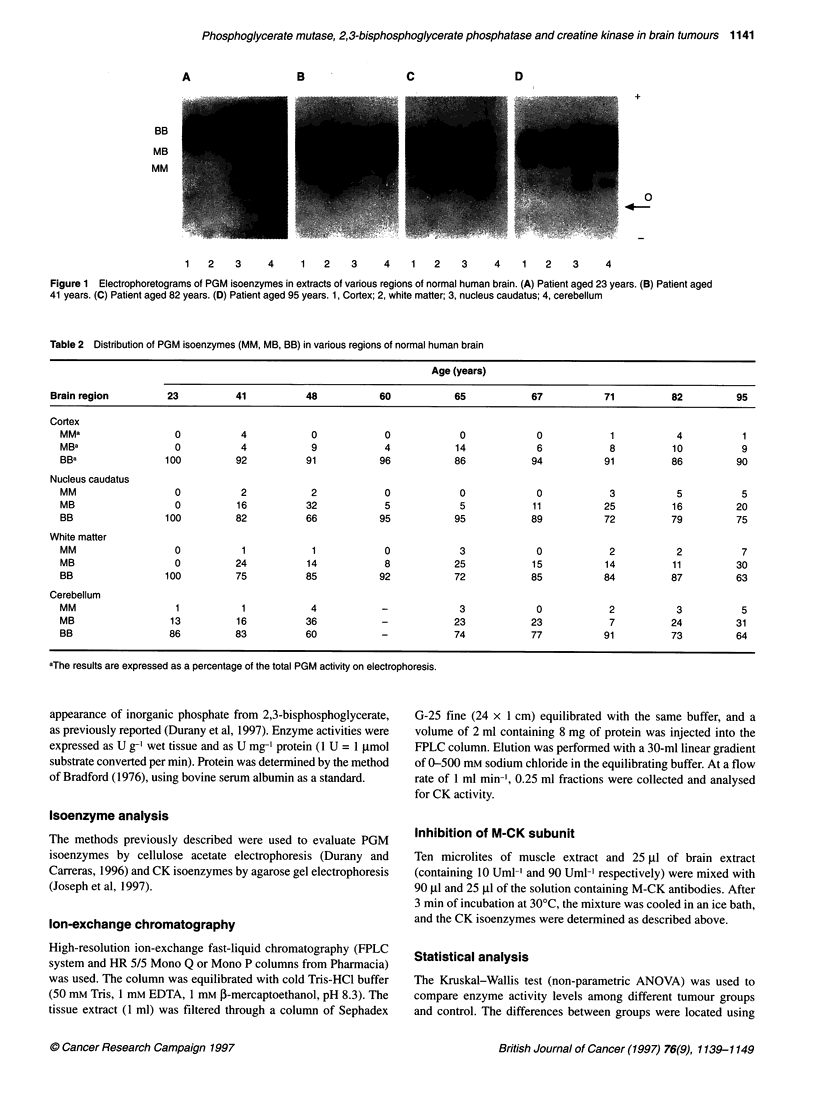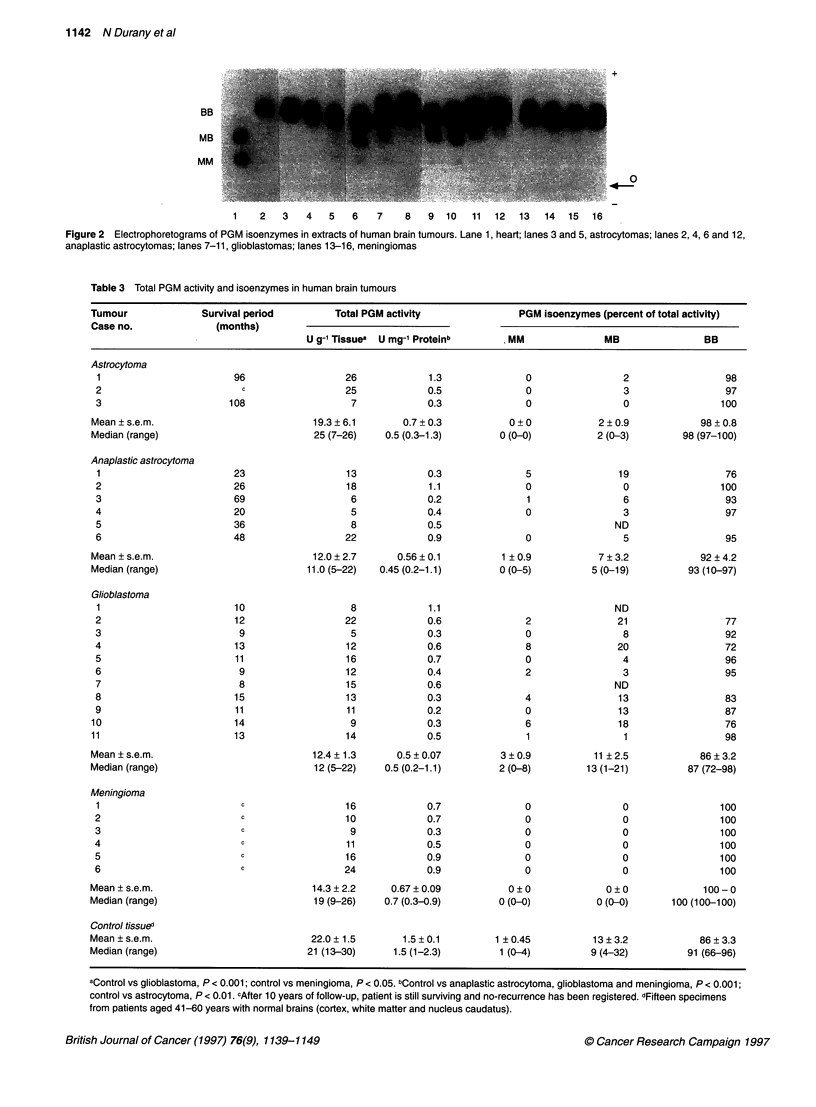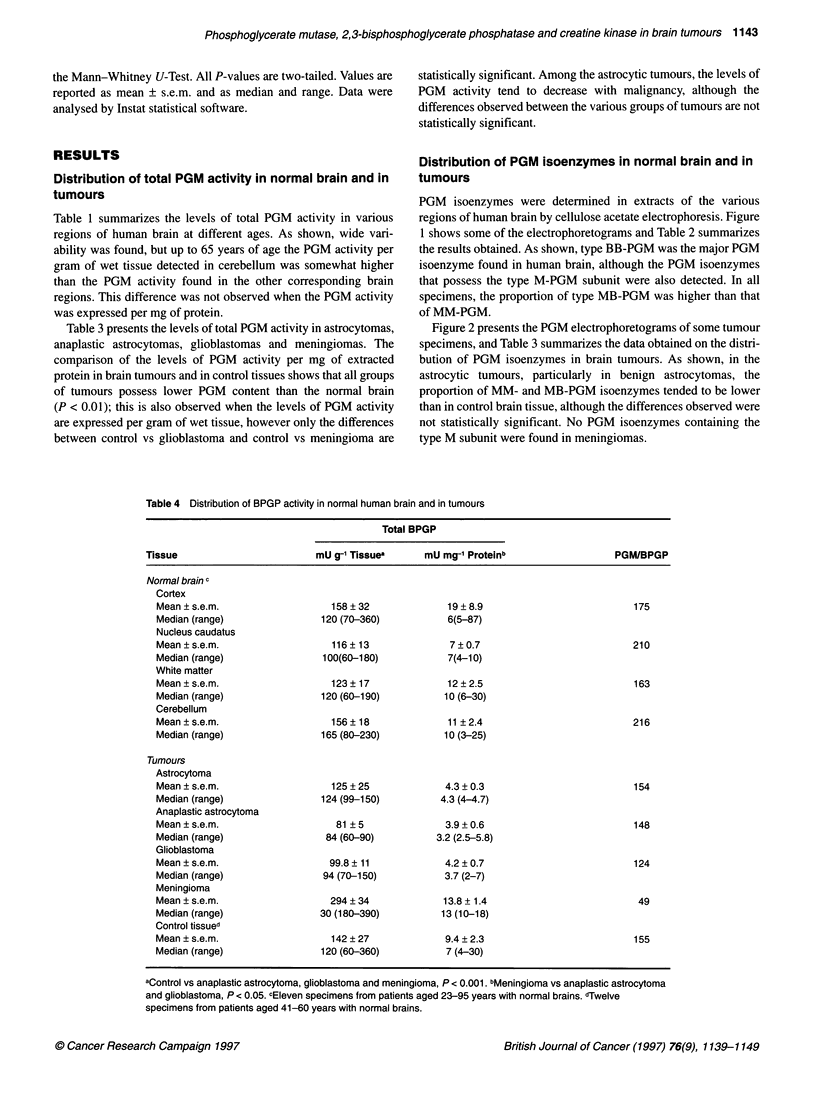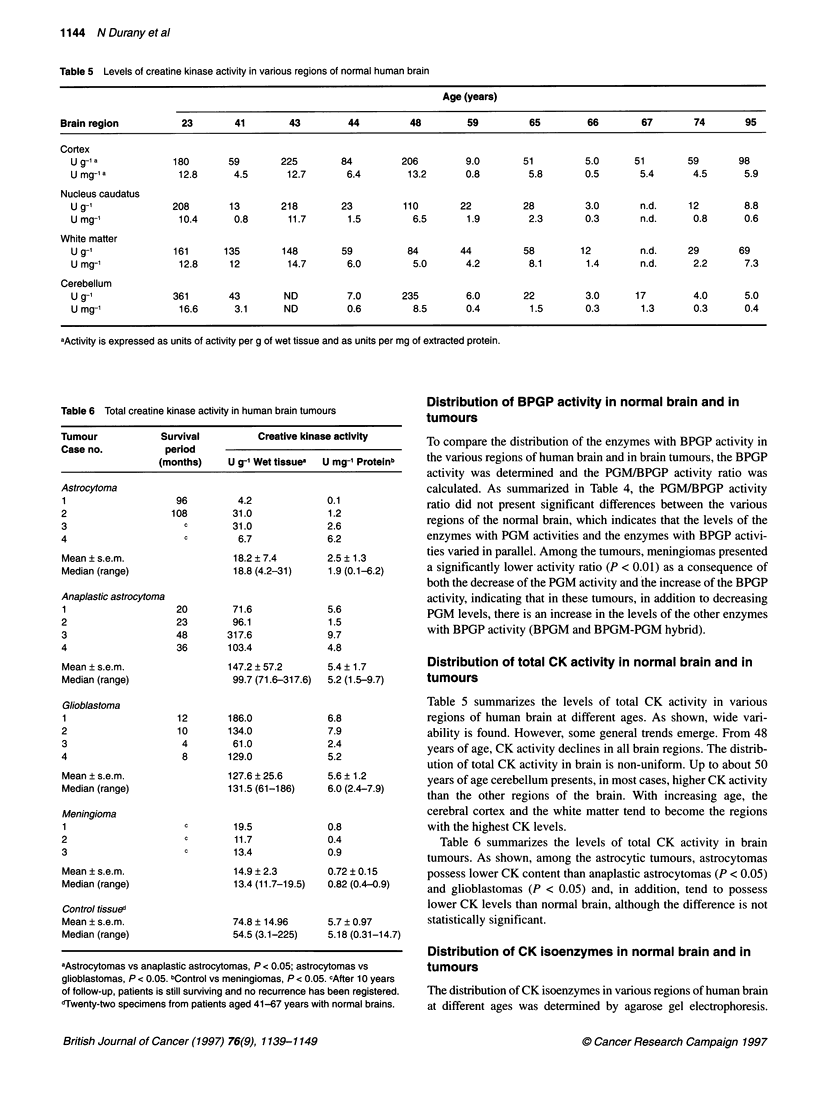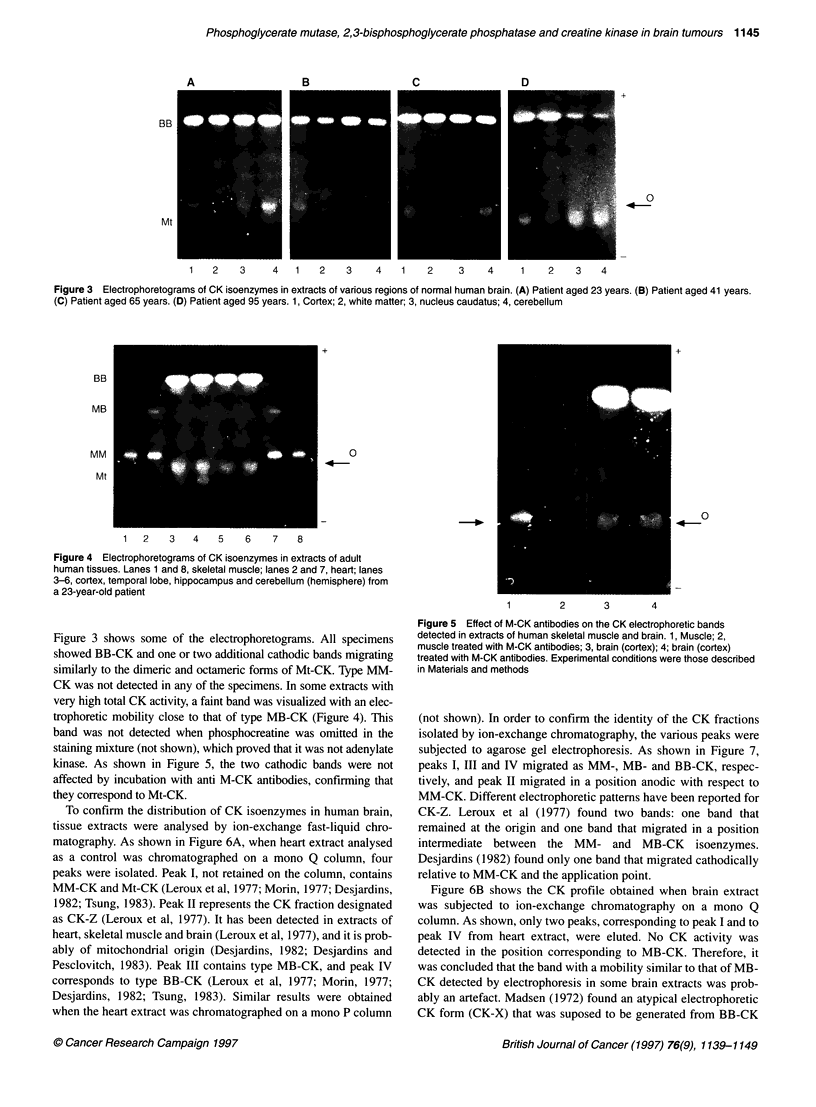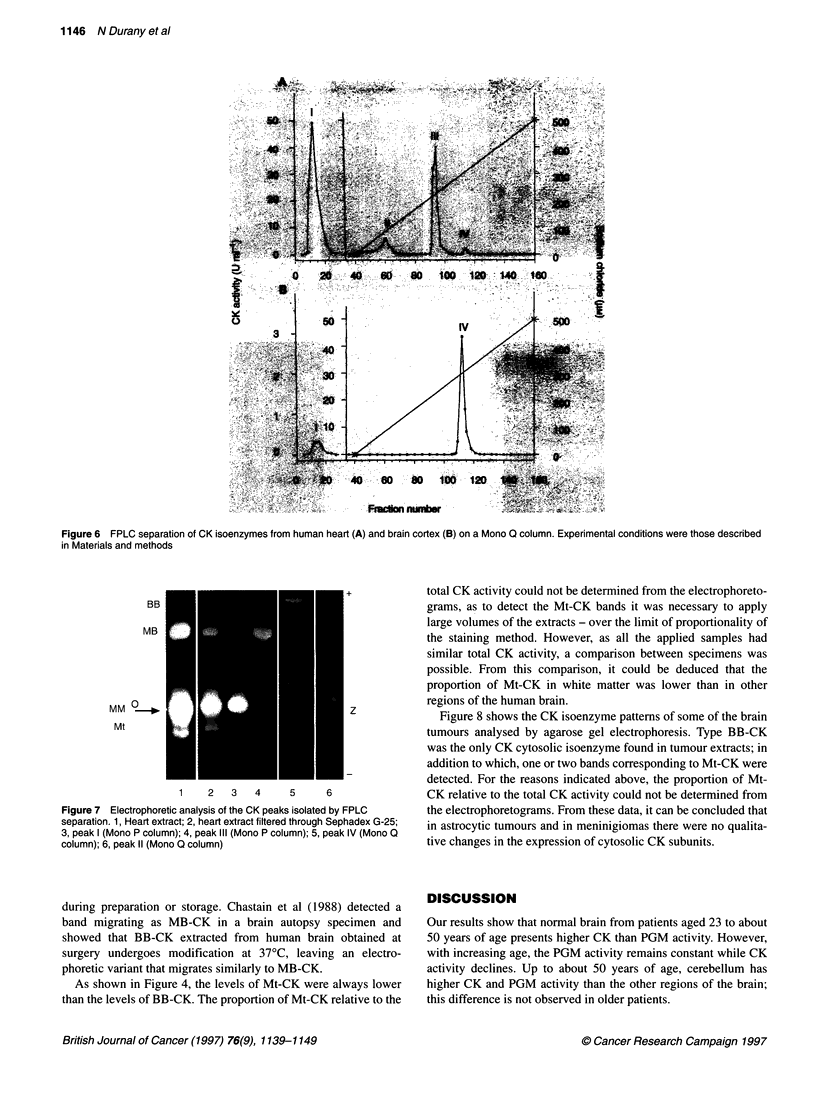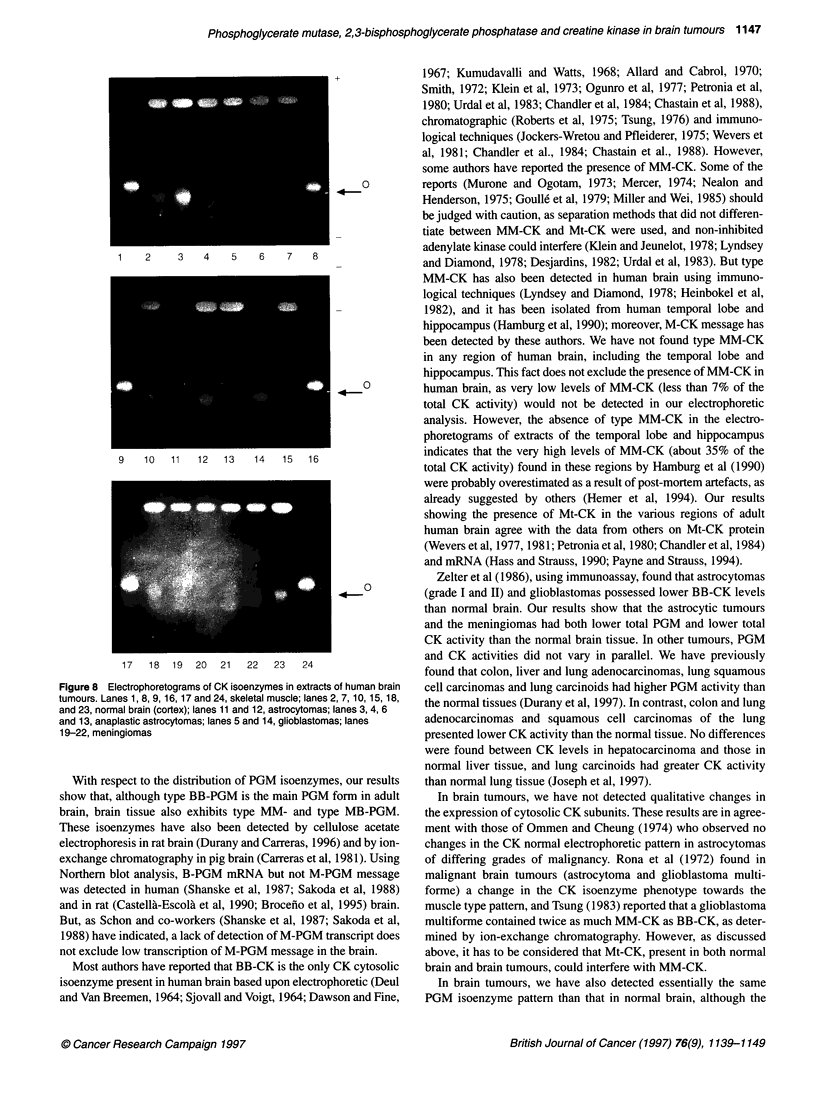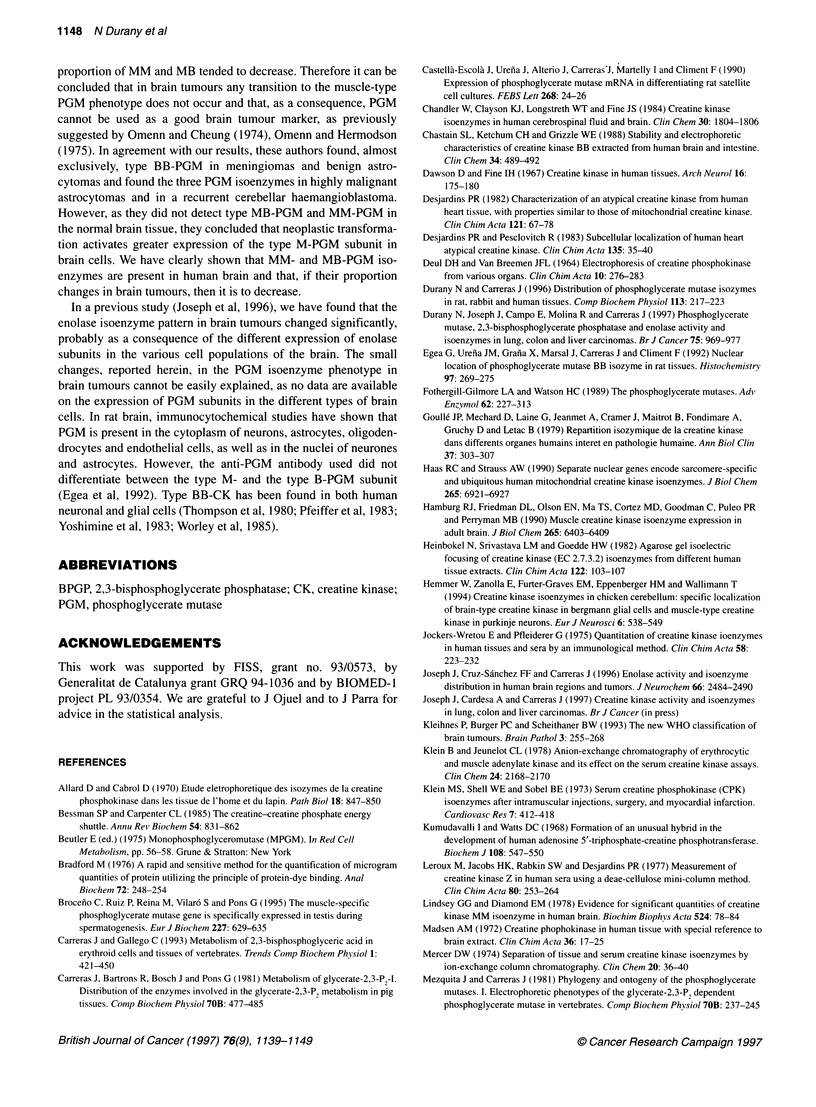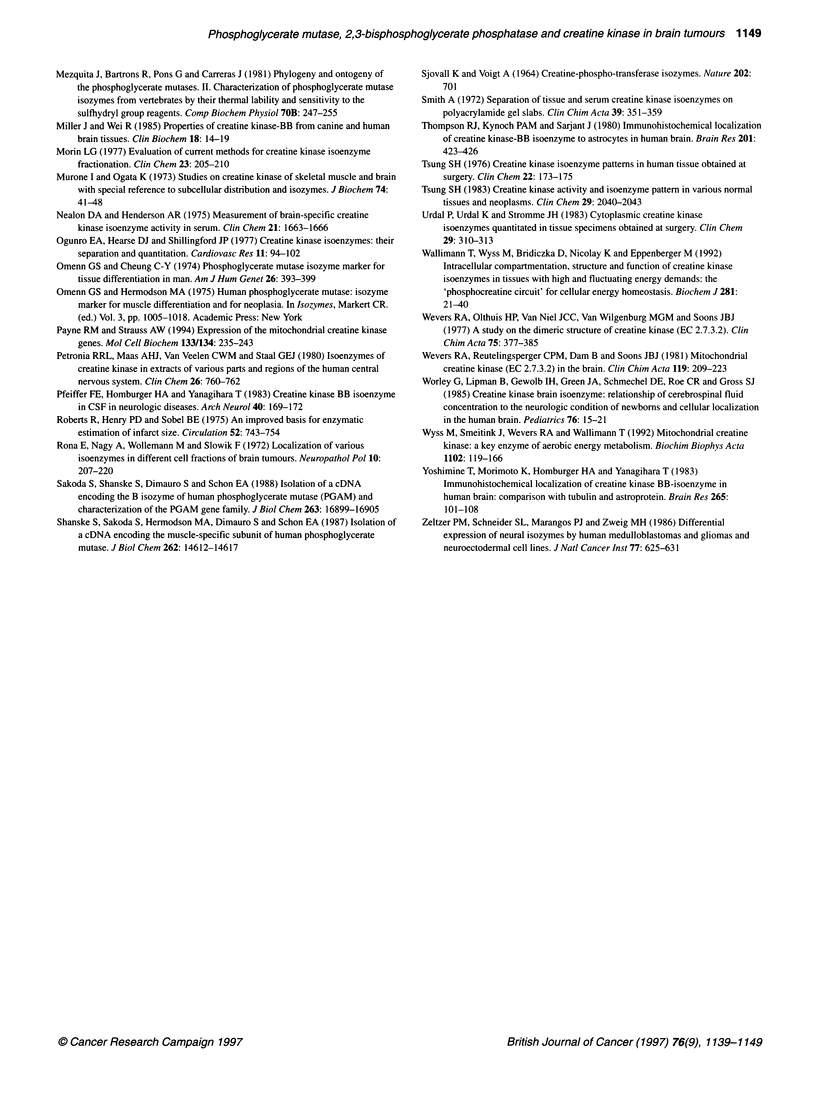# Phosphoglycerate mutase, 2,3-bisphosphoglycerate phosphatase and creatine kinase activity and isoenzymes in human brain tumours.

**DOI:** 10.1038/bjc.1997.525

**Published:** 1997

**Authors:** N. Durany, J. Joseph, F. F. Cruz-SÃ¡nchez, J. Carreras

**Affiliations:** Unit of Biochemistry, Faculty of Medicine, University of Barcelona, Spain.

## Abstract

**Images:**


*British Journal of Cancer* (1997) **76**(9), 1139–1149
© 1997 Cancer Research Campaign

### Phosphoglycerate mutase, 2,3-bisphosphoglycerate phosphatase and creatine kinase activity and isoenzymes in human brain tumours

**N Durany[1], J Joseph[1], FF Cruz-Sánchez[2] and J Carreras[1]**

[1]Unit of Biochemistry, Faculty of Medicine, University of Barcelona, Barcelona, Spain; [2]Neurological Tissue Bank, Department of Medicine (Neurology), Hospital Clínic i Provincial, University of Barcelona, Barcelona, Spain

**Summary** The distribution of phosphoglycerate mutase (EC 5.4.2.1, PGM), 2,3-bisphosphoglycerate phosphatase (EC 3.1.3.13, BPGP) and creatine kinase (EC 2.7.3.2, CK) activity and isoenzymes in various regions of adult human brain and in brain tumours (astrocytomas, anaplastic astrocytomas, glioblastomas and meningiomas) has been determined using electrophoresis. PGM and cytosolic CK exist in mammalian tissues as three isoenzymes that result from the homodimeric and heterodimeric combinations of two subunits [types M (muscle) and B (brain)] coded by separated genes. In addition, a dimeric from and an octameric form of mitochondrial CK exist in mammals. Type BB-PGM was the major PGM isoenzyme found in normal brain, although type MB-PGM and type MM-PGM were also detected. All brain tumours possessed lower PGM activity than normal brain, and meningiomas showed higher BPGP activity. In astrocytic tumours, the proportion of type MB- and type MM-PGM decreased, and in meningiomas these isoenzymes were not detected. Type BB-CK and mitochondrial CK were the only CK isoenzymes detected in normal brain. Astrocytomas possessed lower CK activity than anaplastic astrocytomas and glioblastomas and, in addition, tended to possess lower CK content than normal brain. No qualitative changes of the normal CK isoenzyme pattern were observed in the tumours.

**Keywords:** 2,3-bisphosphoglycerate phosphatase; creatine kinase; phosphoglycerate mutase; activity and isoenzymes; human brain; brain tumour

Most isoenzyme transitions that occur in neoplastic tissues represent a shift from a differentiated to an undifferentiated pattern. The transitions to a more differentiated pattern are much less frequent and involve a great alteration in the control of gene expression. Omenn and co-workers (Omenn and Cheung, 1974; Omenn and Hermodson, 1975) described in human brain tumours a transition in the phosphoglycerate mutase phenotype from the normal brain pattern to a more differentiated muscle-type pattern that correlated with the degree of malignancy of the tumours and that could constitute a good brain tumour marker. As the series of tumours studied by Omenn and Cheung (1974) and Omenn and Hermodson (1975) was small and the distribution of phosphoglycerate mutase isoenzymes in human brain was poorly known, the present study was undertaken. In addition to phosphoglycerate mutase, we have determined creatine kinase, which possesses isoenzymes with a tissue distribution similar to those of phosphoglycerate mutase.

Phosphoglycerate mutase (D-phosphoglycerate 2,3-phosphomutase, EC 5.4.2.1, PGM) is a glycolytic enzyme present in mammalian cells in substantial amounts that catalyses the interconversion of 3-phosphoglycerate and 2-phosphoglycerate in the presence of the cofactor 2,3-bisphosphoglycerate. In addition to the mutase activity, it possesses 2,3-bisphosphoglycerate phosphatase activity stimulated by 2-phosphoglycolate (for a review, see Fothergill-Gilmore and Watson, 1989). Creatine kinase (ATP: creatine *N*-phosphotransferase, EC 2.7.3.2, CK) is an ubiquitous enzyme that functions in the transfer of energy from the mitochondria to the cytosol and that catalyses the reversible transphosphorylation reaction between ATP and creatine, generating ADP and phosphocreatine (for a review, see Bessman and Carpenter, 1985).

In mammals three isoenzymes of PGM and three cytosolic isoenzymes of CK have been detected that result, in both cases, from the homodimeric and the heterodimeric combinations of two different subunits coded by separate genes and designated M (muscle) and B (brain). In early fetal life, type BB-PGM and type BB-CK are the only forms present. During myogenesis the isoenzyme phenotypes undergo transition, type BB-PGM and type BB-CK being replaced by the MM forms through the MB isoenzymes. In adult mammals, skeletal muscle contains almost exclusively type MM-PGM and type MM-CK, whereas type BB-PGM and type BB-CK are found in most types of tissue. Only in heart are the three PGM and CK isoenzymes present in substantial amounts. Mammalian tissues also express two mitochondrial CK (Mt-CK) subunits ('ubiquitous' Mt-CK and 'sarcomeric' or striated muscle-specific Mt-CK) that form octameric and dimeric molecules (for reviews, see Wallimann et al, 1992; Wyss et al, 1992; Carreras and Gallego, 1993; Durany and Carreras, 1996). In addition to PGM isoenzymes, in mammals there are other enzymes that have 2-phosphoglycolate-stimulated BPGP activity. One such enzyme is the 2,3-bisphosphoglycerate synthase phosphatase or 2,3-bisphosphoglycerate mutase (EC 5.4.2.4, BPGM), which is a homodimer of a subunit that possesses great homology with PGM subunits. Two other enzymes are heterodimers resulting from the combination of a BPGM subunit

*Received 22 November 1996*
*Revised 19 March 1997*
*Accepted 2 April 1997*

*Correspondence to:* J Carreras, Unitat de Bioquímica, Facultat de Medicina, Universitat de Barcelona, Casanova, 143, 08036-Barcelona, Spain with a PGM subunit of either type M or type B (for review, see Carreras and Gallego, 1993).

Omenn and co-workers detected only type BB-PGM in human brain (Omenn and Cheung, 1974; Omenn and Herdmodson, 1975), but we have found type MB- and MM-PGM in rat and pig brain, and the hybrid BPGM-type B-PGM in pig brain (Carreras et al, 1981; Mezquita and Carreras, 1981; Mezquita et al, 1981; Durany and Carreras, 1996). Type BB-CK is the major CK cytosolic isoenzyme found in mammalian brain. Whether brain tissue contains significant amounts of type MB-CK and type MM-CK in addition to ubiquitous Mt-CK is controversial (Wallimann et al, 1992; Wyss et al, 1992).

In the present study, we have determined the distribution of the total PGM, BPGP and CK activities and isoenzymes in various regions of human brain at different ages and in brain tumours to identify their changes in neoplastic tissues as a first step to study the alterations of the expression of PGM and CK genes. On the distribution of PGM isoenzymes in brain tumours, only the reports by Omenn and Cheung (1974) and Omenn and Herdmodson (1975) have been published. In addition, there is little information available on CK isoenzymes in brain tumours. Several reports have been published on CK isoenzymes in embryonal brain tumours but, to our knowledge, only three reports exist on the CK isoenzymes phenotype in astrocytic tumours (Rona et al, 1972; Omenn and Cheung, 1974; Tsung, 1983).

### MATERIALS AND METHODS

### Materials

Enzymes, substrates, cofactors and biochemicals were purchased from either Boehringer (Mannheim, Germany) or Sigma (St Louis, MI, USA). CK-MB DS reaction mixture (cat no. 1.12948) from Merck (Darmstadt, Germany) was used as the source of M-CK antibodies. β-Mercaptoethanol was from Merck (Darmstad, Germany). Bovine serum albumin was from Calbiochem (La Jolla, CA, USA). Other chemicals were reagent grade. Agar noble was from Difco Laboratories (Detroit, MI, USA). Agarose gels were from Ciba-Corning (Palo Alto, CA, USA) and cellulose acetate strips were from Helena Laboratories (Beaumont, TX, USA).

### Tissue samples

Brain samples were obtained from the Neurological Tissue Bank, 'Hospital Clinic', University of Barcelona, Spain. Samples from cerebral cortex (superior frontal gyrus), nucleus caudatus (anterior), cerebral white matter (centrum semiovale) and cerebellar hemisphere were used as control. The patients were aged 23, 41, 43, 44, 48, 52, 59, 60, 65, 66, 67, 74, 82 and 95 years, of whom seven were men and seven were women. The post-mortem delay was 1–12 h. Tumour tissues were obtained from material used for biopsy during surgery. Tumours were supratentorial and were classified, according to the WHO brain tumour classification (Kleines et al, 1993), as low-grade astrocytomas (four patients), anaplastic astrocytomas (six patients), glioblastomas (11 patients) and meningiomas (six patients). The mean age of the patients suffering from the tumours was 57.3 years, ranging from 45 to 72 years. Skeletal muscle and heart samples were obtained during autopsy within 24 h after death.

### Tissue extraction

Tissue extracts were prepared by homogenization in three volumes (w/v) of cold 20 mM Tris-HCl buffer, pH 7.5, containing 1 mM EDTA and 1 mM β-mercaptoethanol with a Polytron homogenizer (Lucerne, Switzerland) (position 5, 20 s). Cellular debris was removed by centrifugation at 4°C for 30 min at 12 500 *g*, and the supernatants were used for the assay of enzyme activities and isoenzyme distribution.

### Enzyme and protein assays

All enzymatic activities were measured at 30°C. CK activity was determined by coupling the formation of ATP from ADP and phosphocreatine with the hexokinase- and glucose 6-phosphate dehydrogenase-catalysed reactions, as previously reported (Joseph et al, 1997). PGM activity was determined by coupling the formation of 2-phosphoglycerate from 3-phosphoglycerate with the enolase-, pyruvate kinase- and lactate dehydrogenase-catalysed reactions (Beutler, 1975), as previously described (Durany and Carreras, 1996). BPGP activity was assayed by measuring the

**Table 1** Levels of PGM activity in various regions of normal human brain

| | Age (years) | | | | | | | | | | |
|---|---|---|---|---|---|---|---|---|---|---|---|
| **Brain region** | **23** | **41** | **43** | **48** | **52** | **60** | **65** | **67** | **74** | **82** | **95** |
| Cortex | | | | | | | | | | | |
| U $g^{-1}$ [a] | 21 | 19 | 20 | 30 | 20 | 18 | 20 | 15 | 22 | 27 | 20 |
| U $mg^{-1}$ [a] | 1.1 | 2.3 | 1.1 | 1.6 | 1.9 | 1.1 | 1.8 | 1.0 | 1.4 | 1.2 | 1.4 |
| Nucleus caudatus | | | | | | | | | | | |
| U $g^{-1}$ | 23 | 29 | 21 | 24 | 15 | 18 | 22 | 16 | 23 | 26 | 25 |
| U $mg^{-1}$ | 1.1 | 1.9 | 1.2 | 1.3 | 1.5 | 1.4 | 1.5 | 1.1 | 1.6 | 1.5 | 1.2 |
| White matter | | | | | | | | | | | |
| U $g^{-1}$ | 18 | 24 | 29 | 26 | 13 | 13 | 18 | 17 | 22 | 22 | 18 |
| U $mg^{-1}$ | 1.1 | 1.8 | 2.0 | 1.5 | 1.1 | 1.0 | 1.7 | 1.5 | 1.8 | 1.2 | 1.4 |
| Cerebellum | | | | | | | | | | | |
| U $g^{-1}$ | 25 | 36 | 44 | 42 | 22 | – | 52 | 16 | 16 | 29 | 26 |
| U $mg^{-1}$ | 1.2 | 3.0 | 3.1 | 1.5 | 1.7 | – | 1.9 | 1.3 | 1.3 | 1.0 | 1.4 |

[a]Activity is expressed as units per g of wet tissue and as units per mg of extracted protein.

© Cancer Research Campaign 1997

**Figure 1** Electrophoretograms of PGM isoenzymes in extracts of various regions of normal human brain. (**A**) Patient aged 23 years. (**B**) Patient aged 41 years. (**C**) Patient aged 82 years. (**D**) Patient aged 95 years. 1, Cortex; 2, white matter; 3, nucleus caudatus; 4, cerebellum

**Table 2** Distribution of PGM isoenzymes (MM, MB, BB) in various regions of normal human brain

| | Age (years) | | | | | | | | |
|---|---|---|---|---|---|---|---|---|---|
| **Brain region** | **23** | **41** | **48** | **60** | **65** | **67** | **71** | **82** | **95** |
| Cortex | | | | | | | | | |
| MM[a] | 0 | 4 | 0 | 0 | 0 | 0 | 1 | 4 | 1 |
| MB[a] | 0 | 4 | 9 | 4 | 14 | 6 | 8 | 10 | 9 |
| BB[a] | 100 | 92 | 91 | 96 | 86 | 94 | 91 | 86 | 90 |
| Nucleus caudatus | | | | | | | | | |
| MM | 0 | 2 | 2 | 0 | 0 | 0 | 3 | 5 | 5 |
| MB | 0 | 16 | 32 | 5 | 5 | 11 | 25 | 16 | 20 |
| BB | 100 | 82 | 66 | 95 | 95 | 89 | 72 | 79 | 75 |
| White matter | | | | | | | | | |
| MM | 0 | 1 | 1 | 0 | 3 | 0 | 2 | 2 | 7 |
| MB | 0 | 24 | 14 | 8 | 25 | 15 | 14 | 11 | 30 |
| BB | 100 | 75 | 85 | 92 | 72 | 85 | 84 | 87 | 63 |
| Cerebellum | | | | | | | | | |
| MM | 1 | 1 | 4 | – | 3 | 0 | 2 | 3 | 5 |
| MB | 13 | 16 | 36 | – | 23 | 23 | 7 | 24 | 31 |
| BB | 86 | 83 | 60 | – | 74 | 77 | 91 | 73 | 64 |

[a]The results are expressed as a percentage of the total PGM activity on electrophoresis.

appearance of inorganic phosphate from 2,3-bisphosphoglycerate, as previously reported (Durany et al, 1997). Enzyme activities were expressed as U $g^{-1}$ wet tissue and as U $mg^{-1}$ protein (1 U = 1 μmol substrate converted per min). Protein was determined by the method of Bradford (1976), using bovine serum albumin as a standard.

### Isoenzyme analysis

The methods previously described were used to evaluate PGM isoenzymes by cellulose acetate electrophoresis (Durany and Carreras, 1996) and CK isoenzymes by agarose gel electrophoresis (Joseph et al, 1997).

### Ion-exchange chromatography

High-resolution ion-exchange fast-liquid chromatography (FPLC system and HR 5/5 Mono Q or Mono P columns from Pharmacia) was used. The column was equilibrated with cold Tris-HCl buffer (50 mM Tris, 1 mM EDTA, 1 mM β-mercaptoethanol, pH 8.3). The tissue extract (1 ml) was filtered through a column of Sephadex G-25 fine (24 × 1 cm) equilibrated with the same buffer, and a volume of 2 ml containing 8 mg of protein was injected into the FPLC column. Elution was performed with a 30-ml linear gradient of 0–500 mM sodium chloride in the equilibrating buffer. At a flow rate of 1 ml $min^{-1}$, 0.25 ml fractions were collected and analysed for CK activity.

### Inhibition of M-CK subunit

Ten microlites of muscle extract and 25 μl of brain extract (containing 10 $Uml^{-1}$ and 90 $Uml^{-1}$ respectively) were mixed with 90 μl and 25 μl of the solution containing M-CK antibodies. After 3 min of incubation at 30°C, the mixture was cooled in an ice bath, and the CK isoenzymes were determined as described above.

### Statistical analysis

The Kruskal–Wallis test (non-parametric ANOVA) was used to compare enzyme activity levels among different tumour groups and control. The differences between groups were located using

© Cancer Research Campaign 1997 

**Figure 2** Electrophoretograms of PGM isoenzymes in extracts of human brain tumours. Lane 1, heart; lanes 3 and 5, astrocytomas; lanes 2, 4, 6 and 12, anaplastic astrocytomas; lanes 7–11, glioblastomas; lanes 13–16, meningiomas

**Table 3** Total PGM activity and isoenzymes in human brain tumours

| Tumour Case no. | Survival period (months) | Total PGM activity | | PGM isoenzymes (percent of total activity) | | |
|---|---|---|---|---|---|---|
| | | U $g^{-1}$ Tissue[a] | U $mg^{-1}$ Protein[b] | MM | MB | BB |
| *Astrocytoma* | | | | | | |
| 1 | 96 | 26 | 1.3 | 0 | 2 | 98 |
| 2 | [c] | 25 | 0.5 | 0 | 3 | 97 |
| 3 | 108 | 7 | 0.3 | 0 | 0 | 100 |
| Mean ± s.e.m. | | 19.3 ± 6.1 | 0.7 ± 0.3 | 0 ± 0 | 2 ± 0.9 | 98 ± 0.8 |
| Median (range) | | 25 (7–26) | 0.5 (0.3–1.3) | 0 (0–0) | 2 (0–3) | 98 (97–100) |
| *Anaplastic astrocytoma* | | | | | | |
| 1 | 23 | 13 | 0.3 | 5 | 19 | 76 |
| 2 | 26 | 18 | 1.1 | 0 | 0 | 100 |
| 3 | 69 | 6 | 0.2 | 1 | 6 | 93 |
| 4 | 20 | 5 | 0.4 | 0 | 3 | 97 |
| 5 | 36 | 8 | 0.5 | | ND | |
| 6 | 48 | 22 | 0.9 | 0 | 5 | 95 |
| Mean ± s.e.m. | | 12.0 ± 2.7 | 0.56 ± 0.1 | 1 ± 0.9 | 7 ± 3.2 | 92 ± 4.2 |
| Median (range) | | 11.0 (5–22) | 0.45 (0.2–1.1) | 0 (0–5) | 5 (0–19) | 93 (10–97) |
| *Glioblastoma* | | | | | | |
| 1 | 10 | 8 | 1.1 | | ND | |
| 2 | 12 | 22 | 0.6 | 2 | 21 | 77 |
| 3 | 9 | 5 | 0.3 | 0 | 8 | 92 |
| 4 | 13 | 12 | 0.6 | 8 | 20 | 72 |
| 5 | 11 | 16 | 0.7 | 0 | 4 | 96 |
| 6 | 9 | 12 | 0.4 | 2 | 3 | 95 |
| 7 | 8 | 15 | 0.6 | | ND | |
| 8 | 15 | 13 | 0.3 | 4 | 13 | 83 |
| 9 | 11 | 11 | 0.2 | 0 | 13 | 87 |
| 10 | 14 | 9 | 0.3 | 6 | 18 | 76 |
| 11 | 13 | 14 | 0.5 | 1 | 1 | 98 |
| Mean ± s.e.m. | | 12.4 ± 1.3 | 0.5 ± 0.07 | 3 ± 0.9 | 11 ± 2.5 | 86 ± 3.2 |
| Median (range) | | 12 (5–22) | 0.5 (0.2–1.1) | 2 (0–8) | 13 (1–21) | 87 (72–98) |
| *Meningioma* | | | | | | |
| 1 | [c] | 16 | 0.7 | 0 | 0 | 100 |
| 2 | [c] | 10 | 0.7 | 0 | 0 | 100 |
| 3 | [c] | 9 | 0.3 | 0 | 0 | 100 |
| 4 | [c] | 11 | 0.5 | 0 | 0 | 100 |
| 5 | [c] | 16 | 0.9 | 0 | 0 | 100 |
| 6 | [c] | 24 | 0.9 | 0 | 0 | 100 |
| Mean ± s.e.m. | | 14.3 ± 2.2 | 0.67 ± 0.09 | 0 ± 0 | 0 ± 0 | 100 – 0 |
| Median (range) | | 19 (9–26) | 0.7 (0.3–0.9) | 0 (0–0) | 0 (0–0) | 100 (100–100) |
| *Control tissue*[d] | | | | | | |
| Mean ± s.e.m. | | 22.0 ± 1.5 | 1.5 ± 0.1 | 1 ± 0.45 | 13 ± 3.2 | 86 ± 3.3 |
| Median (range) | | 21 (13–30) | 1.5 (1–2.3) | 1 (0–4) | 9 (4–32) | 91 (66–96) |

[a]Control vs glioblastoma, $P < 0.001$; control vs meningioma, $P < 0.05$. [b]Control vs anaplastic astrocytoma, glioblastoma and meningioma, $P < 0.001$; control vs astrocytoma, $P < 0.01$. [c]After 10 years of follow-up, patient is still surviving and no-recurrence has been registered. [d]Fifteen specimens from patients aged 41–60 years with normal brains (cortex, white matter and nucleus caudatus).

 © Cancer Research Campaign 1997

the Mann–Whitney *U*-Test. All *P*-values are two-tailed. Values are reported as mean ± s.e.m. and as median and range. Data were analysed by Instat statistical software.

### RESULTS

### Distribution of total PGM activity in normal brain and in tumours

Table 1 summarizes the levels of total PGM activity in various regions of human brain at different ages. As shown, wide variability was found, but up to 65 years of age the PGM activity per gram of wet tissue detected in cerebellum was somewhat higher than the PGM activity found in the other corresponding brain regions. This difference was not observed when the PGM activity was expressed per mg of protein.

Table 3 presents the levels of total PGM activity in astrocytomas, anaplastic astrocytomas, glioblastomas and meningiomas. The comparison of the levels of PGM activity per mg of extracted protein in brain tumours and in control tissues shows that all groups of tumours possess lower PGM content than the normal brain ($P < 0.01$); this is also observed when the levels of PGM activity are expressed per gram of wet tissue, however only the differences between control vs glioblastoma and control vs meningioma are statistically significant. Among the astrocytic tumours, the levels of PGM activity tend to decrease with malignancy, although the differences observed between the various groups of tumours are not statistically significant.

### Distribution of PGM isoenzymes in normal brain and in tumours

PGM isoenzymes were determined in extracts of the various regions of human brain by cellulose acetate electrophoresis. Figure 1 shows some of the electrophoretograms and Table 2 summarizes the results obtained. As shown, type BB-PGM was the major PGM isoenzyme found in human brain, although the PGM isoenzymes that possess the type M-PGM subunit were also detected. In all specimens, the proportion of type MB-PGM was higher than that of MM-PGM.

Figure 2 presents the PGM electrophoretograms of some tumour specimens, and Table 3 summarizes the data obtained on the distribution of PGM isoenzymes in brain tumours. As shown, in the astrocytic tumours, particularly in benign astrocytomas, the proportion of MM- and MB-PGM isoenzymes tended to be lower than in control brain tissue, although the differences observed were not statistically significant. No PGM isoenzymes containing the type M subunit were found in meningiomas.

**Table 4** Distribution of BPGP activity in normal human brain and in tumours

| Tissue | Total BPGP mU $g^{-1}$ Tissue[a] | Total BPGP mU $mg^{-1}$ Protein[b] | PGM/BPGP |
|---|---|---|---|
| *Normal brain* [c] | | | |
| Cortex | | | |
| Mean ± s.e.m. | 158 ± 32 | 19 ± 8.9 | 175 |
| Median (range) | 120 (70–360) | 6(5–87) | |
| Nucleus caudatus | | | |
| Mean ± s.e.m. | 116 ± 13 | 7 ± 0.7 | 210 |
| Median (range) | 100(60–180) | 7(4–10) | |
| White matter | | | |
| Mean ± s.e.m. | 123 ± 17 | 12 ± 2.5 | 163 |
| Median (range) | 120 (60–190) | 10 (6–30) | |
| Cerebellum | | | |
| Mean ± s.e.m. | 156 ± 18 | 11 ± 2.4 | 216 |
| Median (range) | 165 (80–230) | 10 (3–25) | |
| *Tumours* | | | |
| Astrocytoma | | | |
| Mean ± s.e.m. | 125 ± 25 | 4.3 ± 0.3 | 154 |
| Median (range) | 124 (99–150) | 4.3 (4–4.7) | |
| Anaplastic astrocytoma | | | |
| Mean ± s.e.m. | 81 ± 5 | 3.9 ± 0.6 | 148 |
| Median (range) | 84 (60–90) | 3.2 (2.5–5.8) | |
| Glioblastoma | | | |
| Mean ± s.e.m. | 99.8 ± 11 | 4.2 ± 0.7 | 124 |
| Median (range) | 94 (70–150) | 3.7 (2–7) | |
| Meningioma | | | |
| Mean ± s.e.m. | 294 ± 34 | 13.8 ± 1.4 | 49 |
| Median (range) | 30 (180–390) | 13 (10–18) | |
| Control tissue[d] | | | |
| Mean ± s.e.m. | 142 ± 27 | 9.4 ± 2.3 | 155 |
| Median (range) | 120 (60–360) | 7 (4–30) | |

[a]Control vs anaplastic astrocytoma, glioblastoma and meningioma, $P < 0.001$. [b]Meningioma vs anaplastic astrocytoma and glioblastoma, $P < 0.05$. [c]Eleven specimens from patients aged 23–95 years with normal brains. [d]Twelve specimens from patients aged 41–60 years with normal brains.

© Cancer Research Campaign 1997

Table 5 Levels of creatine kinase activity in various regions of normal human brain

| Brain region | Age (years) 23 | 41 | 43 | 44 | 48 | 59 | 65 | 66 | 67 | 74 | 95 |
|---|---|---|---|---|---|---|---|---|---|---|---|
| Cortex | | | | | | | | | | | |
| U g$^{-1}$ [a] | 180 | 59 | 225 | 84 | 206 | 9.0 | 51 | 5.0 | 51 | 59 | 98 |
| U mg$^{-1}$ [a] | 12.8 | 4.5 | 12.7 | 6.4 | 13.2 | 0.8 | 5.8 | 0.5 | 5.4 | 4.5 | 5.9 |
| Nucleus caudatus | | | | | | | | | | | |
| U g$^{-1}$ | 208 | 13 | 218 | 23 | 110 | 22 | 28 | 3.0 | n.d. | 12 | 8.8 |
| U mg$^{-1}$ | 10.4 | 0.8 | 11.7 | 1.5 | 6.5 | 1.9 | 2.3 | 0.3 | n.d. | 0.8 | 0.6 |
| White matter | | | | | | | | | | | |
| U g$^{-1}$ | 161 | 135 | 148 | 59 | 84 | 44 | 58 | 12 | n.d. | 29 | 69 |
| U mg$^{-1}$ | 12.8 | 12 | 14.7 | 6.0 | 5.0 | 4.2 | 8.1 | 1.4 | n.d. | 2.2 | 7.3 |
| Cerebellum | | | | | | | | | | | |
| U g$^{-1}$ | 361 | 43 | ND | 7.0 | 235 | 6.0 | 22 | 3.0 | 17 | 4.0 | 5.0 |
| U mg$^{-1}$ | 16.6 | 3.1 | ND | 0.6 | 8.5 | 0.4 | 1.5 | 0.3 | 1.3 | 0.3 | 0.4 |

[a]Activity is expressed as units of activity per g of wet tissue and as units per mg of extracted protein.

Table 6 Total creatine kinase activity in human brain tumours

| Tumour Case no. | Survival period (months) | Creative kinase activity U g$^{-1}$ Wet tissue[a] | U mg$^{-1}$ Protein[b] |
|---|---|---|---|
| *Astrocytoma* | | | |
| 1 | 96 | 4.2 | 0.1 |
| 2 | 108 | 31.0 | 1.2 |
| 3 | [c] | 31.0 | 2.6 |
| 4 | [c] | 6.7 | 6.2 |
| Mean ± s.e.m. | | 18.2 ± 7.4 | 2.5 ± 1.3 |
| Median (range) | | 18.8 (4.2–31) | 1.9 (0.1–6.2) |
| *Anaplastic astrocytoma* | | | |
| 1 | 20 | 71.6 | 5.6 |
| 2 | 23 | 96.1 | 1.5 |
| 3 | 48 | 317.6 | 9.7 |
| 4 | 36 | 103.4 | 4.8 |
| Mean ± s.e.m. | | 147.2 ± 57.2 | 5.4 ± 1.7 |
| Median (range) | | 99.7 (71.6–317.6) | 5.2 (1.5–9.7) |
| *Glioblastoma* | | | |
| 1 | 12 | 186.0 | 6.8 |
| 2 | 10 | 134.0 | 7.9 |
| 3 | 4 | 61.0 | 2.4 |
| 4 | 8 | 129.0 | 5.2 |
| Mean ± s.e.m. | | 127.6 ± 25.6 | 5.6 ± 1.2 |
| Median (range) | | 131.5 (61–186) | 6.0 (2.4–7.9) |
| *Meningioma* | | | |
| 1 | [c] | 19.5 | 0.8 |
| 2 | [c] | 11.7 | 0.4 |
| 3 | [c] | 13.4 | 0.9 |
| Mean ± s.e.m. | | 14.9 ± 2.3 | 0.72 ± 0.15 |
| Median (range) | | 13.4 (11.7–19.5) | 0.82 (0.4–0.9) |
| *Control tissue*[d] | | | |
| Mean ± s.e.m. | | 74.8 ± 14.96 | 5.7 ± 0.97 |
| Median (range) | | 54.5 (3.1–225) | 5.18 (0.31–14.7) |

[a]Astrocytomas vs anaplastic astrocytomas, $P < 0.05$; astrocytomas vs glioblastomas, $P < 0.05$. [b]Control vs meningiomas, $P < 0.05$. [c]After 10 years of follow-up, patients is still surviving and no recurrence has been registered. [d]Twenty-two specimens from patients aged 41–67 years with normal brains.

### Distribution of BPGP activity in normal brain and in tumours

To compare the distribution of the enzymes with BPGP activity in the various regions of human brain and in brain tumours, the BPGP activity was determined and the PGM/BPGP activity ratio was calculated. As summarized in Table 4, the PGM/BPGP activity ratio did not present significant differences between the various regions of the normal brain, which indicates that the levels of the enzymes with PGM activities and the enzymes with BPGP activities varied in parallel. Among the tumours, meningiomas presented a significantly lower activity ratio ($P < 0.01$) as a consequence of both the decrease of the PGM activity and the increase of the BPGP activity, indicating that in these tumours, in addition to decreasing PGM levels, there is an increase in the levels of the other enzymes with BPGP activity (BPGM and BPGM-PGM hybrid).

### Distribution of total CK activity in normal brain and in tumours

Table 5 summarizes the levels of total CK activity in various regions of human brain at different ages. As shown, wide variability is found. However, some general trends emerge. From 48 years of age, CK activity declines in all brain regions. The distribution of total CK activity in brain is non-uniform. Up to about 50 years of age cerebellum presents, in most cases, higher CK activity than the other regions of the brain. With increasing age, the cerebral cortex and the white matter tend to become the regions with the highest CK levels.

Table 6 summarizes the levels of total CK activity in brain tumours. As shown, among the astrocytic tumours, astrocytomas possess lower CK content than anaplastic astrocytomas ($P < 0.05$) and glioblastomas ($P < 0.05$) and, in addition, tend to possess lower CK levels than normal brain, although the difference is not statistically significant.

### Distribution of CK isoenzymes in normal brain and in tumours

The distribution of CK isoenzymes in various regions of human brain at different ages was determined by agarose gel electrophoresis.

© Cancer Research Campaign 1997

**Figure 3** Electrophoretograms of CK isoenzymes in extracts of various regions of normal human brain. (**A**) Patient aged 23 years. (**B**) Patient aged 41 years. (**C**) Patient aged 65 years. (**D**) Patient aged 95 years. 1, Cortex; 2, white matter; 3, nucleus caudatus; 4, cerebellum

**Figure 4** Electrophoretograms of CK isoenzymes in extracts of adult human tissues. Lanes 1 and 8, skeletal muscle; lanes 2 and 7, heart; lanes 3–6, cortex, temporal lobe, hippocampus and cerebellum (hemisphere) from a 23-year-old patient

**Figure 5** Effect of M-CK antibodies on the CK electrophoretic bands detected in extracts of human skeletal muscle and brain. 1, Muscle; 2, muscle treated with M-CK antibodies; 3, brain (cortex); 4; brain (cortex) treated with M-CK antibodies. Experimental conditions were those described in Materials and methods

Figure 3 shows some of the electrophoretograms. All specimens showed BB-CK and one or two additional cathodic bands migrating similarly to the dimeric and octameric forms of Mt-CK. Type MM-CK was not detected in any of the specimens. In some extracts with very high total CK activity, a faint band was visualized with an electrophoretic mobility close to that of type MB-CK (Figure 4). This band was not detected when phosphocreatine was omitted in the staining mixture (not shown), which proved that it was not adenylate kinase. As shown in Figure 5, the two cathodic bands were not affected by incubation with anti M-CK antibodies, confirming that they correspond to Mt-CK.

To confirm the distribution of CK isoenzymes in human brain, tissue extracts were analysed by ion-exchange fast-liquid chromatography. As shown in Figure 6A, when heart extract analysed as a control was chromatographed on a mono Q column, four peaks were isolated. Peak I, not retained on the column, contains MM-CK and Mt-CK (Leroux et al, 1977; Morin, 1977; Desjardins, 1982; Tsung, 1983). Peak II represents the CK fraction designated as CK-Z (Leroux et al, 1977). It has been detected in extracts of heart, skeletal muscle and brain (Leroux et al, 1977), and it is probably of mitochondrial origin (Desjardins, 1982; Desjardins and Pesclovitch, 1983). Peak III contains type MB-CK, and peak IV corresponds to type BB-CK (Leroux et al, 1977; Morin, 1977; Desjardins, 1982; Tsung, 1983). Similar results were obtained when the heart extract was chromatographed on a mono P column (not shown). In order to confirm the identity of the CK fractions isolated by ion-exchange chromatography, the various peaks were subjected to agarose gel electrophoresis. As shown in Figure 7, peaks I, III and IV migrated as MM-, MB- and BB-CK, respectively, and peak II migrated in a position anodic with respect to MM-CK. Different electrophoretic patterns have been reported for CK-Z. Leroux et al (1977) found two bands: one band that remained at the origin and one band that migrated in a position intermediate between the MM- and MB-CK isoenzymes. Desjardins (1982) found only one band that migrated cathodically relative to MM-CK and the application point.

Figure 6B shows the CK profile obtained when brain extract was subjected to ion-exchange chromatography on a mono Q column. As shown, only two peaks, corresponding to peak I and to peak IV from heart extract, were eluted. No CK activity was detected in the position corresponding to MB-CK. Therefore, it was concluded that the band with a mobility similar to that of MB-CK detected by electrophoresis in some brain extracts was probably an artefact. Madsen (1972) found an atypical electrophoretic CK form (CK-X) that was suposed to be generated from BB-CK

© Cancer Research Campaign 1997

**Figure 6** FPLC separation of CK isoenzymes from human heart (**A**) and brain cortex (**B**) on a Mono Q column. Experimental conditions were those described in Materials and methods

**Figure 7** Electrophoretic analysis of the CK peaks isolated by FPLC separation. 1, Heart extract; 2, heart extract filtered through Sephadex G-25; 3, peak I (Mono P column); 4, peak III (Mono P column); 5, peak IV (Mono Q column); 6, peak II (Mono Q column)

during preparation or storage. Chastain et al (1988) detected a band migrating as MB-CK in a brain autopsy specimen and showed that BB-CK extracted from human brain obtained at surgery undergoes modification at 37°C, leaving an electrophoretic variant that migrates similarly to MB-CK.

As shown in Figure 4, the levels of Mt-CK were always lower than the levels of BB-CK. The proportion of Mt-CK relative to the total CK activity could not be determined from the electrophoretograms, as to detect the Mt-CK bands it was necessary to apply large volumes of the extracts – over the limit of proportionality of the staining method. However, as all the applied samples had similar total CK activity, a comparison between specimens was possible. From this comparison, it could be deduced that the proportion of Mt-CK in white matter was lower than in other regions of the human brain.

Figure 8 shows the CK isoenzyme patterns of some of the brain tumours analysed by agarose gel electrophoresis. Type BB-CK was the only CK cytosolic isoenzyme found in tumour extracts; in addition to which, one or two bands corresponding to Mt-CK were detected. For the reasons indicated above, the proportion of Mt-CK relative to the total CK activity could not be determined from the electrophoretograms. From these data, it can be concluded that in astrocytic tumours and in meninigiomas there were no qualitative changes in the expression of cytosolic CK subunits.

### DISCUSSION

Our results show that normal brain from patients aged 23 to about 50 years of age presents higher CK than PGM activity. However, with increasing age, the PGM activity remains constant while CK activity declines. Up to about 50 years of age, cerebellum has higher CK and PGM activity than the other regions of the brain; this difference is not observed in older patients.

*© Cancer Research Campaign 1997*

**Figure 8** Electrophoretograms of CK isoenzymes in extracts of human brain tumours. Lanes 1, 8, 9, 16, 17 and 24, skeletal muscle; lanes 2, 7, 10, 15, 18, and 23, normal brain (cortex); lanes 11 and 12, astrocytomas; lanes 3, 4, 6 and 13, anaplastic astrocytomas; lanes 5 and 14, glioblastomas; lanes 19–22, meningiomas

With respect to the distribution of PGM isoenzymes, our results show that, although type BB-PGM is the main PGM form in adult brain, brain tissue also exhibits type MM- and type MB-PGM. These isoenzymes have also been detected by cellulose acetate electrophoresis in rat brain (Durany and Carreras, 1996) and by ion-exchange chromatography in pig brain (Carreras et al, 1981). Using Northern blot analysis, B-PGM mRNA but not M-PGM message was detected in human (Shanske et al, 1987; Sakoda et al, 1988) and in rat (Castellà-Escolà et al, 1990; Broceño et al, 1995) brain. But, as Schon and co-workers (Shanske et al, 1987; Sakoda et al, 1988) have indicated, a lack of detection of M-PGM transcript does not exclude low transcription of M-PGM message in the brain.

Most authors have reported that BB-CK is the only CK cytosolic isoenzyme present in human brain based upon electrophoretic (Deul and Van Breemen, 1964; Sjovall and Voigt, 1964; Dawson and Fine, 1967; Kumudavalli and Watts, 1968; Allard and Cabrol, 1970; Smith, 1972; Klein et al, 1973; Ogunro et al, 1977; Petronia et al, 1980; Urdal et al, 1983; Chandler et al, 1984; Chastain et al, 1988), chromatographic (Roberts et al, 1975; Tsung, 1976) and immunological techniques (Jockers-Wretou and Pfleiderer, 1975; Wevers et al, 1981; Chandler et al., 1984; Chastain et al., 1988). However, some authors have reported the presence of MM-CK. Some of the reports (Murone and Ogotam, 1973; Mercer, 1974; Nealon and Henderson, 1975; Goullé et al, 1979; Miller and Wei, 1985) should be judged with caution, as separation methods that did not differentiate between MM-CK and Mt-CK were used, and non-inhibited adenylate kinase could interfere (Klein and Jeunelot, 1978; Lyndsey and Diamond, 1978; Desjardins, 1982; Urdal et al, 1983). But type MM-CK has also been detected in human brain using immunological techniques (Lyndsey and Diamond, 1978; Heinbokel et al, 1982), and it has been isolated from human temporal lobe and hippocampus (Hamburg et al, 1990); moreover, M-CK message has been detected by these authors. We have not found type MM-CK in any region of human brain, including the temporal lobe and hippocampus. This fact does not exclude the presence of MM-CK in human brain, as very low levels of MM-CK (less than 7% of the total CK activity) would not be detected in our electrophoretic analysis. However, the absence of type MM-CK in the electrophoretograms of extracts of the temporal lobe and hippocampus indicates that the very high levels of MM-CK (about 35% of the total CK activity) found in these regions by Hamburg et al (1990) were probably overestimated as a result of post-mortem artefacts, as already suggested by others (Hemer et al, 1994). Our results showing the presence of Mt-CK in the various regions of adult human brain agree with the data from others on Mt-CK protein (Wevers et al, 1977, 1981; Petronia et al, 1980; Chandler et al, 1984) and mRNA (Hass and Strauss, 1990; Payne and Strauss, 1994).

Zelter et al (1986), using immunoassay, found that astrocytomas (grade I and II) and glioblastomas possessed lower BB-CK levels than normal brain. Our results show that the astrocytic tumours and the meningiomas had both lower total PGM and lower total CK activity than the normal brain tissue. In other tumours, PGM and CK activities did not vary in parallel. We have previously found that colon, liver and lung adenocarcinomas, lung squamous cell carcinomas and lung carcinoids had higher PGM activity than the normal tissues (Durany et al, 1997). In contrast, colon and lung adenocarcinomas and squamous cell carcinomas of the lung presented lower CK activity than the normal tissue. No differences were found between CK levels in hepatocarcinoma and those in normal liver tissue, and lung carcinoids had greater CK activity than normal lung tissue (Joseph et al, 1997).

In brain tumours, we have not detected qualitative changes in the expression of cytosolic CK subunits. These results are in agreement with those of Ommen and Cheung (1974) who observed no changes in the CK normal electrophoretic pattern in astrocytomas of differing grades of malignancy. Rona et al (1972) found in malignant brain tumours (astrocytoma and glioblastoma multiforme) a change in the CK isoenzyme phenotype towards the muscle type pattern, and Tsung (1983) reported that a glioblastoma multiforme contained twice as much MM-CK as BB-CK, as determined by ion-exchange chromatography. However, as discussed above, it has to be considered that Mt-CK, present in both normal brain and brain tumours, could interfere with MM-CK.

In brain tumours, we have also detected essentially the same PGM isoenzyme pattern than that in normal brain, although the

© Cancer Research Campaign 1997

proportion of MM and MB tended to decrease. Therefore it can be concluded that in brain tumours any transition to the muscle-type PGM phenotype does not occur and that, as a consequence, PGM cannot be used as a good brain tumour marker, as previously suggested by Omenn and Cheung (1974), Omenn and Hermodson (1975). In agreement with our results, these authors found, almost exclusively, type BB-PGM in meningiomas and benign astrocytomas and found the three PGM isoenzymes in highly malignant astrocytomas and in a recurrent cerebellar haemangioblastoma. However, as they did not detect type MB-PGM and MM-PGM in the normal brain tissue, they concluded that neoplastic transformation activates greater expression of the type M-PGM subunit in brain cells. We have clearly shown that MM- and MB-PGM isoenzymes are present in human brain and that, if their proportion changes in brain tumours, then it is to decrease.

In a previous study (Joseph et al, 1996), we have found that the enolase isoenzyme pattern in brain tumours changed significantly, probably as a consequence of the different expression of enolase subunits in the various cell populations of the brain. The small changes, reported herein, in the PGM isoenzyme phenotype in brain tumours cannot be easily explained, as no data are available on the expression of PGM subunits in the different types of brain cells. In rat brain, immunocytochemical studies have shown that PGM is present in the cytoplasm of neurons, astrocytes, oligodendrocytes and endothelial cells, as well as in the nuclei of neurones and astrocytes. However, the anti-PGM antibody used did not differentiate between the type M- and the type B-PGM subunit (Egea et al, 1992). Type BB-CK has been found in both human neuronal and glial cells (Thompson et al, 1980; Pfeiffer et al, 1983; Yoshimine et al, 1983; Worley et al, 1985).

### ABBREVIATIONS

BPGP, 2,3-bisphosphoglycerate phosphatase; CK, creatine kinase; PGM, phosphoglycerate mutase

### ACKNOWLEDGEMENTS

This work was supported by FISS, grant no. 93/0573, by Generalitat de Catalunya grant GRQ 94-1036 and by BIOMED-1 project PL 93/0354. We are grateful to J Ojuel and to J Parra for advice in the statistical analysis.

### REFERENCES

Allard D and Cabrol D (1970) Etude eletrophoretique des isozymes de la creatine phosphokinase dans les tissue de l'home et du lapin. *Path Biol* **18**: 847–850

Bessman SP and Carpenter CL (1985) The creatine–creatine phosphate energy shuttle. *Annu Rev Biochem* **54**: 831–862

Beutler E (ed.) (1975) Monophosphoglyceromutase (MPGM). In *Red Cell Metabolism*, pp. 56–58. Grune & Stratton: New York

Bradford M (1976) A rapid and sensitive method for the quantification of microgram quantities of protein utilizing the principle of protein-dye binding. *Anal Biochem* **72**: 248–254

Broceño C, Ruiz P, Reina M, Vilaró S and Pons G (1995) The muscle-specific phosphoglycerate mutase gene is specifically expressed in testis during spermatogenesis. *Eur J Biochem* **227**: 629–635

Carreras J and Gallego C (1993) Metabolism of 2,3-bisphosphoglyceric acid in erythroid cells and tissues of vertebrates. *Trends Comp Biochem Physiol* **1**: 421–450

Carreras J, Bartrons R, Bosch J and Pons G (1981) Metabolism of glycerate-2,3-$P_2$-I. Distribution of the enzymes involved in the glycerate-2,3-$P_2$ metabolism in pig tissues. *Comp Biochem Physiol* **70B**: 477–485

Castellà-Escolà J, Ureña J, Alterio J, Carreras J, Martelly I and Climent F (1990) Expression of phosphoglycerate mutase mRNA in differentiating rat satellite cell cultures. *FEBS Lett* **268**: 24–26

Chandler W, Clayson KJ, Longstreth WT and Fine JS (1984) Creatine kinase isoenzymes in human cerebrospinal fluid and brain. *Clin Chem* **30**: 1804–1806

Chastain SL, Ketchum CH and Grizzle WE (1988) Stability and electrophoretic characteristics of creatine kinase BB extracted from human brain and intestine. *Clin Chem* **34**: 489–492

Dawson D and Fine IH (1967) Creatine kinase in human tissues. *Arch Neurol* **16**: 175–180

Desjardins PR (1982) Characterization of an atypical creatine kinase from human heart tissue, with properties similar to those of mitochondrial creatine kinase. *Clin Chim Acta* **121**: 67–78

Desjardins PR and Pesclovitch R (1983) Subcellular localization of human heart atypical creatine kinase. *Clin Chim Acta* **135**: 35–40

Deul DH and Van Breemen JFL (1964) Electrophoresis of creatine phosphokinase from various organs. *Clin Chim Acta* **10**: 276–283

Durany N and Carreras J (1996) Distribution of phosphoglycerate mutase isozymes in rat, rabbit and human tissues. *Comp Biochem Physiol* **113**: 217–223

Durany N, Joseph J, Campo E, Molina R and Carreras J (1997) Phosphoglycerate mutase, 2,3-bisphosphoglycerate phosphatase and enolase activity and isoenzymes in lung, colon and liver carcinomas. *Br J Cancer* **75**: 969–977

Egea G, Ureña JM, Graña X, Marsal J, Carreras J and Climent F (1992) Nuclear location of phosphoglycerate mutase BB isozyme in rat tissues. *Histochemistry* **97**: 269–275

Fothergill-Gilmore LA and Watson HC (1989) The phosphoglycerate mutases. *Adv Enzymol* **62**: 227–313

Goullé JP, Mechard D, Laine G, Jeanmet A, Cramer J, Maitrot B, Fondimare A, Gruchy D and Letac B (1979) Repartition isozymique de la creatine kinase dans differents organes humains interet en pathologie humaine. *Ann Biol Clin* **37**: 303–307

Haas RC and Strauss AW (1990) Separate nuclear genes encode sarcomere-specific and ubiquitous human mitochondrial creatine kinase isoenzymes. *J Biol Chem* **265**: 6921–6927

Hamburg RJ, Friedman DL, Olson EN, Ma TS, Cortez MD, Goodman C, Puleo PR and Perryman MB (1990) Muscle creatine kinase isoenzyme expression in adult brain. *J Biol Chem* **265**: 6403–6409

Heinbokel N, Srivastava LM and Goedde HW (1982) Agarose gel isoelectric focusing of creatine kinase (EC 2.7.3.2) isoenzymes from different human tissue extracts. *Clin Chim Acta* **122**: 103–107

Hemmer W, Zanolla E, Furter-Graves EM, Eppenberger HM and Wallimann T (1994) Creatine kinase isoenzymes in chicken cerebellum: specific localization of brain-type creatine kinase in bergmann glial cells and muscle-type creatine kinase in purkinje neurons. *Eur J Neurosci* **6**: 538–549

Jockers-Wretou E and Pfleiderer G (1975) Quantitation of creatine kinase ioenzymes in human tissues and sera by an immunological method. *Clin Chim Acta* **58**: 223–232

Joseph J, Cruz-Sánchez FF and Carreras J (1996) Enolase activity and isoenzyme distribution in human brain regions and tumors. *J Neurochem* **66**: 2484–2490

Joseph J, Cardesa A and Carreras J (1997) Creatine kinase activity and isoenzymes in lung, colon and liver carcinomas. *Br J Cancer* (in press)

Kleihnes P, Burger PC and Scheithaner BW (1993) The new WHO classification of brain tumours. *Brain Pathol* **3**: 255–268

Klein B and Jeunelot CL (1978) Anion-exchange chromatography of erythrocytic and muscle adenylate kinase and its effect on the serum creatine kinase assays. *Clin Chem* **24**: 2168–2170

Klein MS, Shell WE and Sobel BE (1973) Serum creatine phosphokinase (CPK) isoenzymes after intramuscular injections, surgery, and myocardial infarction. *Cardiovasc Res* **7**: 412–418

Kumudavalli I and Watts DC (1968) Formation of an unusual hybrid in the development of human adenosine 5′-triphosphate-creatine phosphotransferase. *Biochem J* **108**: 547–550

Leroux M, Jacobs HK, Rabkin SW and Desjardins PR (1977) Measurement of creatine kinase Z in human sera using a deae-cellulose mini-column method. *Clin Chim Acta* **80**: 253–264

Lindsey GG and Diamond EM (1978) Evidence for significant quantities of creatine kinase MM isoenzyme in human brain. *Biochim Biophys Acta* **524**: 78–84

Madsen AM (1972) Creatine phophokinase in human tissue with special reference to brain extract. *Clin Chim Acta* **36**: 17–25

Mercer DW (1974) Separation of tissue and serum creatine kinase isoenzymes by ion-exchange column chromatography. *Clin Chem* **20**: 36–40

Mezquita J and Carreras J (1981) Phylogeny and ontogeny of the phosphoglycerate mutases. I. Electrophoretic phenotypes of the glycerate-2,3-$P_2$ dependent phosphoglycerate mutase in vertebrates. *Comp Biochem Physiol* **70B**: 237–245

 © Cancer Research Campaign 1997

Mezquita J, Bartrons R, Pons G and Carreras J (1981) Phylogeny and ontogeny of the phosphoglycerate mutases. II. Characterization of phosphoglycerate mutase isozymes from vertebrates by their thermal lability and sensitivity to the sulfhydryl group reagents. *Comp Biochem Physiol* **70B**: 247–255

Miller J and Wei R (1985) Properties of creatine kinase-BB from canine and human brain tissues. *Clin Biochem* **18**: 14–19

Morin LG (1977) Evaluation of current methods for creatine kinase isoenzyme fractionation. *Clin Chem* **23**: 205–210

Murone I and Ogata K (1973) Studies on creatine kinase of skeletal muscle and brain with special reference to subcellular distribution and isozymes. *J Biochem* **74**: 41–48

Nealon DA and Henderson AR (1975) Measurement of brain-specific creatine kinase isoenzyme activity in serum. *Clin Chem* **21**: 1663–1666

Ogunro EA, Hearse DJ and Shillingford JP (1977) Creatine kinase isoenzymes: their separation and quantitation. *Cardiovasc Res* **11**: 94–102

Omenn GS and Cheung C-Y (1974) Phosphoglycerate mutase isozyme marker for tissue differentiation in man. *Am J Hum Genet* **26**: 393–399

Omenn GS and Hermodson MA (1975) Human phosphoglycerate mutase: isozyme marker for muscle differentiation and for neoplasia. In *Isozymes*, Markert CR. (ed.) Vol. 3, pp. 1005–1018. Academic Press: New York

Payne RM and Strauss AW (1994) Expression of the mitochondrial creatine kinase genes. *Mol Cell Biochem* **133/134**: 235–243

Petronia RRL, Maas AHJ, Van Veelen CWM and Staal GEJ (1980) Isoenzymes of creatine kinase in extracts of various parts and regions of the human central nervous system. *Clin Chem* **26**: 760–762

Pfeiffer FE, Homburger HA and Yanagihara T (1983) Creatine kinase BB isoenzyme in CSF in neurologic diseases. *Arch Neurol* **40**: 169–172

Roberts R, Henry PD and Sobel BE (1975) An improved basis for enzymatic estimation of infarct size. *Circulation* **52**: 743–754

Rona E, Nagy A, Wollemann M and Slowik F (1972) Localization of various isoenzymes in different cell fractions of brain tumours. *Neuropathol Pol* **10**: 207–220

Sakoda S, Shanske S, Dimauro S and Schon EA (1988) Isolation of a cDNA encoding the B isozyme of human phosphoglycerate mutase (PGAM) and characterization of the PGAM gene family. *J Biol Chem* **263**: 16899–16905

Shanske S, Sakoda S, Hermodson MA, Dimauro S and Schon EA (1987) Isolation of a cDNA encoding the muscle-specific subunit of human phosphoglycerate mutase. *J Biol Chem* **262**: 14612–14617

Sjovall K and Voigt A (1964) Creatine-phospho-transferase isozymes. *Nature* **202**: 701

Smith A (1972) Separation of tissue and serum creatine kinase isoenzymes on polyacrylamide gel slabs. *Clin Chim Acta* **39**: 351–359

Thompson RJ, Kynoch PAM and Sarjant J (1980) Immunohistochemical localization of creatine kinase-BB isoenzyme to astrocytes in human brain. *Brain Res* **201**: 423–426

Tsung SH (1976) Creatine kinase isoenzyme patterns in human tissue obtained at surgery. *Clin Chem* **22**: 173–175

Tsung SH (1983) Creatine kinase activity and isoenzyme pattern in various normal tissues and neoplasms. *Clin Chem* **29**: 2040–2043

Urdal P, Urdal K and Stromme JH (1983) Cytoplasmic creatine kinase isoenzymes quantitated in tissue specimens obtained at surgery. *Clin Chem* **29**: 310–313

Wallimann T, Wyss M, Bridiczka D, Nicolay K and Eppenberger M (1992) Intracellular compartmentation, structure and function of creatine kinase isoenzymes in tissues with high and fluctuating energy demands: the 'phosphocreatine circuit' for cellular energy homeostasis. *Biochem J* **281**: 21–40

Wevers RA, Olthuis HP, Van Niel JCC, Van Wilgenburg MGM and Soons JBJ (1977) A study on the dimeric structure of creatine kinase (EC 2.7.3.2). *Clin Chim Acta* **75**: 377–385

Wevers RA, Reutelingsperger CPM, Dam B and Soons JBJ (1981) Mitochondrial creatine kinase (EC 2.7.3.2) in the brain. *Clin Chim Acta* **119**: 209–223

Worley G, Lipman B, Gewolb IH, Green JA, Schmechel DE, Roe CR and Gross SJ (1985) Creatine kinase brain isoenzyme: relationship of cerebrospinal fluid concentration to the neurologic condition of newborns and cellular localization in the human brain. *Pediatrics* **76**: 15–21

Wyss M, Smeitink J, Wevers RA and Wallimann T (1992) Mitochondrial creatine kinase: a key enzyme of aerobic energy metabolism. *Biochim Biophys Acta* **1102**: 119–166

Yoshimine T, Morimoto K, Homburger HA and Yanagihara T (1983) Immunohistochemical localization of creatine kinase BB-isoenzyme in human brain: comparison with tubulin and astroprotein. *Brain Res* **265**: 101–108

Zeltzer PM, Schneider SL, Marangos PJ and Zweig MH (1986) Differential expression of neural isozymes by human medulloblastomas and gliomas and neuroectodermal cell lines. *J Natl Cancer Inst* **77**: 625–631

© Cancer Research Campaign 1997